# No evidence that frailty modifies the positive impact of antihypertensive treatment in very elderly people: an investigation of the impact of frailty upon treatment effect in the HYpertension in the Very Elderly Trial (HYVET) study, a double-blind, placebo-controlled study of antihypertensives in people with hypertension aged 80 and over

**DOI:** 10.1186/s12916-015-0328-1

**Published:** 2015-04-09

**Authors:** Jane Warwick, Emanuela Falaschetti, Kenneth Rockwood, Arnold Mitnitski, Lutgarde Thijs, Nigel Beckett, Christopher Bulpitt, Ruth Peters

**Affiliations:** Warwick Clinical Trials Unit, Division of Health Sciences, Warwick Medical School, University of Warwick, Gibbet Hill Campus, Coventry, CV4 7AL UK; Imperial Clinical Trials Unit, School of Public Health, Faculty of Medicine, Imperial College London, St Mary’s Campus, Norfolk Place, London W2 1PG UK; Geriatric Medicine, Dalhousie University, Queen Elizabeth II Health Sciences Centre, 1421-5955 Veterans’ Memorial Lane, Halifax, B3H 1C6 Nova Scotia Canada; Department of Medicine, Dalhousie University, Centre for Health Care of the Elderly, 1421-5955 Veterans’ Memorial Lane, Halifax, B3H 2E1 Nova Scotia Canada; Studies Coordinating Centre, Research Unit Hypertension and Cardiovascular Epidemiology, Department of Cardiovascular Sciences, University of Leuven, Campus Sint Rafaël, Kapucijnenvoer 35, Box 7001, BE-3000 Leuven, Belgium; Department of Medicine, Imperial College London, Hammersmith Campus, London, W12 0NN UK

**Keywords:** Ageing, Antihypertensives, Frailty, Hypertension

## Abstract

**Background:**

Treatment for hypertension with antihypertensive medication has been shown to reduce stroke, cardiovascular events, and mortality in older adults, but there is concern that such treatment may not be appropriate in frailer older adults. To investigate whether there is an interaction between effect of treatment for hypertension and frailty in older adults, we calculated the frailty index (FI) for all available participants from the HYpertension in the Very Elderly Trial (HYVET) study, a double-blind, placebo-controlled study of antihypertensives in people with hypertension aged 80 and over, and obtained frailty adjusted estimates of the effect of treatment with antihypertensive medication on risk of stroke, cardiovascular events, and mortality.

**Methods:**

Participants in HYVET were randomised 1:1 to active treatment with indapamide sustained release 1.5 mg ± perindopril 2 to 4 mg or to matching placebo. Data relating to blood pressure, comorbidities, cognitive function, depression, and quality of life were collected at entry into the study and at subsequent follow-up visits. The FI was calculated at entry, based on 60 potential deficits. The distribution of FI was similar to that seen in population studies of adults aged 80 years and above (median FI, 0.17; IQR, 0.11–0.24). Cox regression was used to assess the impact of FI at entry to the study on subsequent risk of stroke, total mortality, and cardiovascular events. Models were stratified by region of recruitment and adjusted for sex and age at entry. Extending these models to include a term for a possible interaction between treatment for hypertension and FI provided a formula for the treatment effect as a function of FI. For all three models, the point estimates of the hazard ratios for the treatment effect decreased as FI increased, although to varying degrees and with varying certainty.

**Results:**

We found no evidence of an interaction between effect of treatment for hypertension and frailty as measured by the FI. Both the frailer and the fitter older adults with hypertension appeared to gain from treatment.

**Conclusions:**

Further work to examine whether antihypertensive treatment modifies frailty as measured by the FI should be explored.

**Trial registration:**

ClinicalTrials.gov NCT00122811 (July 2005)

**Electronic supplementary material:**

The online version of this article (doi:10.1186/s12916-015-0328-1) contains supplementary material, which is available to authorized users.

## Background

The global population is ageing and with ageing comes an increased prevalence of both frailty and hypertension [1-3]. Treatment for hypertension with antihypertensive medication has been shown to reduce stroke, cardiovascular events, and mortality in older adults [4,5]. Nevertheless, the potential benefit associated with providing treatment for hypertension must be weighed against the potential risk of overtreatment (excessive blood pressure lowering), polypharmacy, and the impact of side effects. There is concern that the treatment may not be beneficial in all older adults, particularly the frailest [6].

Frailty, a clinical state in which there is an increase in an individual’s vulnerability for developing increased dependency and/or mortality when exposed to a stressor [7], has been assessed in a number of ways, varying from evaluation of specific impairments [8] to a holistic operational definition, the Frailty Index (FI), which is quantified by counting the number of diseases, symptoms, or similar health ‘deficits’ [3,9]. The FI theoretically ranges from 0 to 1.0, with a higher FI indicating a higher level of frailty, but in practice has a maximum observed value of around 0.7 and, perhaps because it is more comprehensive, has been shown to be a better predictor of new disability and mortality than the Cardiovascular Health Study or Study of Osteoporotic Fractures scales [10]. FI may be calculated from any data set with sufficient information related to participant deficits (the recommended minimum is 30 deficits that are not saturated within the data set) and in a number of global population datasets has shown consistent relationships between ageing and mortality, with higher frailty scores at older ages and higher frailty associated with an increased risk of death [11-16]. The deficits included in the FI do not need to be independent of each other. Population studies of very old adults report a mean FI of around 0.16 to 18 in those in their early 80s rising to approximately 0.20 over 90 years [13,17]. The FI is applicable at any age, but of particular use in very elderly people, in whom frailty levels are more widely distributed than in the general population, i.e., ranging from the very frail to the robust fitter older adult [17].

The HYpertension in the Very Elderly Trial (HYVET) was a double-blind, placebo-controlled study of antihypertensives in people with hypertension aged 80 and over, which found that treatment with antihypertensives would lead to a reduction in risk of stroke, cardiovascular events, and total mortality [4,5]. However, participants recruited to HYVET, in common with many clinical trials and other studies, are likely to have been healthier than the general very elderly hypertensive population [18]. In consequence, the applicability of the results to the wider elderly population has been questioned, so that uncertainty remains as to whether treatment benefits also extend to the frailer elderly people [6]. To investigate further, we calculated the FI for all available HYVET study participants and obtained frailty-adjusted estimates of the effect of antihypertensive treatment in very elderly people.

## Methods

The HYVET trial randomised participants 1:1 to active treatment with indapamide sustained release 1.5 mg ± perindopril 2 to 4 mg or to matching placebo. The full inclusion criteria for entry into HYVET have been reported in full elsewhere [4]; briefly, participants were required to be hypertensive (average sitting systolic blood pressure ≥160 mmHg), to have no condition likely to limit life to less than a year, no diagnosis of dementia, and no need of 24-hour nursing care. Participants gave written informed consent and were assessed at baseline and at 3-monthly intervals for the first year and 6-monthly intervals thereafter until they either died, withdrew from the study, or the trial ended. Data relating to blood pressure, comorbidities, cognitive function, depression, and quality of life were collected at baseline and at subsequent follow-up visits. Key relevant endpoints were selected prior to the start of the trial and these included incident stroke (the primary endpoint), total mortality, and incident cardiovascular events. Data relating to trial endpoints were collected as these occurred and the endpoints validated by an independent blinded committee. All appropriate ethical and regulatory permissions were obtained. Trial registration number is NCT00122811.

The FI was calculated at entry to the study and based on 60 deficits as detailed in Additional file 1. Each deficit was coded as either present or absent in accordance with previous published methodology [19]. To be eligible for inclusion in the FI, the risk of developing a deficit must increase in the general population with increasing age, be associated with varied organ systems, have negative health associations, and be present in at least roughly 1% of the population under study but not to be saturated in the study data [19]. The number of deficits present was counted for each study participant and divided by 60, the maximum number of deficits possible in our data, to give the FI. Information on all 60 deficits was not available for all participants, partly because some had opted out of the quality of life sub-study and partly because of the usual missing data issues. Where missing data meant that the FI calculation for a particular participant would be based on fewer than the intended 60 possible deficits, the FI was calculated as the number of deficits divided by the number available for that participant. Where missing data meant that the FI calculation for a particular participant would be based on fewer than the recommended minimum of 30 possible deficits, the FI was not calculated but set to missing and the subject excluded from the analysis.

Cox regression [20] was used to assess the impact of FI at entry to the study on subsequent risk of fatal and non-fatal stroke, total mortality, and cardiovascular events (fatal and non-fatal stroke, myocardial infarction and heart failure). Models were stratified by region of recruitment (Western Europe, Eastern Europe, China) to allow each to have a different baseline hazard function (calibrate FI) and adjusted for sex and age at entry to the study. There was no adjustment for baseline cardiovascular disease as this forms part of the FI. The validity of the proportional hazards assumption was assessed using diagnostic plots and Grambsch and Therneau tests [21], and the overall model fit was assessed graphically by plotting the Nelson-Aalen cumulative hazard function versus the Cox-Snell residuals and comparing to a 45° reference line.

We fitted Cox regression models (stratified by region) with terms for baseline age, sex, FI, treatment, and an interaction between treatment and FI to obtain a formula for the treatment effect as a function of FI [22] to illustrate the impact of FI upon the estimate of treatment effect obtained from our models. The estimates of treatment effect from this model, and associated 95% confidence intervals (CIs), were then presented graphically.

The relationship between baseline FI and subsequent drop out was investigated to establish whether participants for whom the FI could not be calculated, owing to incomplete data, differed substantially from those for whom the FI could be calculated.

## Results

### Characteristics of participants

The HYVET trial randomised 3,845 participants, of whom only the 2,656 who consented to complete an additional quality of life questionnaire provided sufficient data to allow the calculation of the FI. The baseline characteristics of these participants are given in Table 1. There did not appear to be any imbalance between the treatment groups.

**Table 1:** **Baseline characteristics of the 2,656 participants of HYVET for whom the frailty index was calculable, by treatment group**

	**Placebo**	**Active**
n	1,324	1,332
Age (yrs), mean (SD)	83.4 (3.0)	83.6 (3.2)
Male	520 (39.3%)	526 (39.5%)
Female	804 (60.7%)	806 (60.5%)
Body mass index*		
Underweight	39 (3%)	58 (4%)
Normal weight	587 (44%)	605 (46%)
Overweight	566 (43%)	530 (40%)
Obese	132 (10%)	138 (10%)
Sitting SBP, mean (SD)	173.1 (8.9)	173.3 (8.8)
Sitting DBP, mean (SD)	90.0 (8.9)	89.9 (8.8)
Standing SBP, mean (SD)	168.0 (11.8)	168.2 (11.9)
Standing DBP, mean (SD)	87.9 (9.9)	88.1 (9.8)
Cardiovascular disease, n (%)	177 (13.4%)	159 (11.9%)
Antihypertensive treatment prior to entry into the trial, n (%)	830 (62.7%)	828 (62.2%)
Mini Mental State Examination, median (IQR)	26.0 (22–28)	26.0 (22–28)
Frailty Index, median (IQR)	0.17 (0.11–0.24)	0.16 (0.11–0.24)

*Adjusted for region of recruitment.

DBP, Diastolic blood pressure; IQR, Interquartile range; SBP, Systolic blood pressure; SD, Standard deviation.

We also compared those for whom the FI was calculable with those for whom it was not. There was no difference between them in terms of age, sex, previous cardiovascular disease, or baseline sitting systolic blood pressure but there was a difference in cognitive test score; the median Mini-Mental State Exam (MMSE) score at entry to the study in those in whom FI was calculable was 26.0 (Interquartile range (IQR), 22–28) and in those in whom FI was not calculable was 27.0 (IQR, 24–29) (*P* <0.001, Mann–Witney test).

### Frailty index (FI)

**Figure 1:**
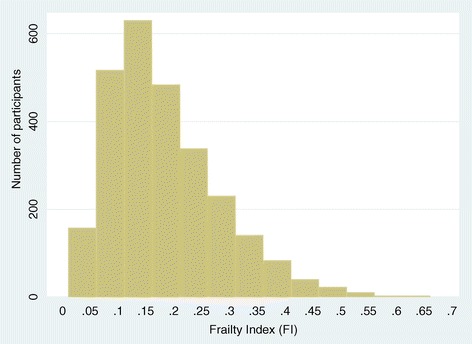
Histogram showing the distribution of frailty index (FI) among 2,656 participants of HYVET at entry to the study.

The distribution of the constituents used to calculate the FI was similar in the two treatment groups (Additional file 2: Table S1). The distribution of FI at entry to the study was skewed with mean and median values 0.19 (standard deviation (SD), 0.10) and 0.17 (IQR, 0.11–0.24), respectively, and range 0.01 to 0.63 (Figure 1). Median FI was higher (*P* <0.01, Mann–Whitney rank-sum test) for women (0.18; IQR, 0.12–0.26) compared to men (0.15; IQR, 0.10–0.21). On average, FI scores increased slowly with age (0.003 per year of age (95% CI, 0.002–0.004)). Greater FI at entry to the study was associated with an increased risk of death (HR, 1.24 per 0.05 increase in FI; 95% CI, 1.18–1.30), cardiovascular events (HR, 1.23 per 0.05 increase in FI; 95% CI, 1.16–1.30), and stroke (HR, 1.26 per 0.05 increase in FI; 95% CI, 1.15–1.37). Adjustment for treatment group, age, and sex, and stratification by region of recruitment did not alter these findings.

### Effect of frailty on estimates of treatment effect from HYVET

Median follow-up was similar in the two treatment groups. For time to death, median follow-up was 22 months (IQR, 13–34) in the placebo group compared to 23 months (IQR, 13–35) in the active treatment group; for time to cardiovascular events, median follow-up was 21 months in the placebo group (IQR, 12–33) and 23 months (IQR, 13–35) in the active treatment group; and for time to stroke, median follow-up was 21 months (IQR, 12–34) in the placebo group and 23 months (IQR, 13–34) in the active treatment group. The proportionality assumption was not violated (*P* values for the global Grambsch and Therneau tests of the proportional hazards assumption for the models for time to death, time to cardiovascular events, and time to stroke were 0.78, 0.14, and 0.39, respectively) and overall model fit was adequate. The estimate of treatment effect obtained from the Cox regression models (Table 2) did not change with adjustment for baseline FI. Treatment with an anti-hypertensive was associated with a 36% reduction in risk of fatal and non-fatal stroke (HR, 0.64; 95% CI, 0.42–0.96; *P* = 0.03) after adjustment for baseline FI, sex, and age, and stratification for region of recruitment. Similarly, active treatment within HYVET significantly reduced the risk of fatal and non-fatal cardiovascular events (HR, 0.59; 95% CI, 0.45–0.77) but there was no significant difference in total mortality between the placebo and active treatment groups (HR, 0.83; 95% CI, 0.66–1.04). The adjusted hazard ratios (HR) for all three endpoints were similar to those seen in the main trial results (which were HR, 0.68; 95% CI, 0.47–0.98 for stroke; HR, 0.66; 95% CI, 0.53–0.82 for cardiovascular events, and HR, 0.79; 95% CI, 0.65–0.95 for mortality [23]). Repeating these analyses with previous cardiovascular disease excluded from the calculation of FI did not materially affect the results.

**Table 2:** **Hazard ratios and associated 95% confidence intervals from Cox regression models showing the effect of adjusting for frailty on the estimate of treatment effect in those for whom a frailty index was calculated n = 2,656**

**Variables included in the model**	**Stroke (95 events)**	**Cardiovascular events (231 events)**	**Total mortality (294 events)**
Treatment group	0.65 (0.43–0.98)	0.59 (0.45–0.77)	0.83 (0.66–1.05)
Treatment group, sex, and age	0.65 (0.43–0.98)	0.59 (0.45–0.77)	0.83 (0.66–1.05)
Treatment group, sex, age, and FI at entry to the study	0.64 (0.42–0.96)	0.59 (0.45–0.77)	0.83 (0.66–1.04)

All models stratified by region of recruitment.

**Figure 2:**
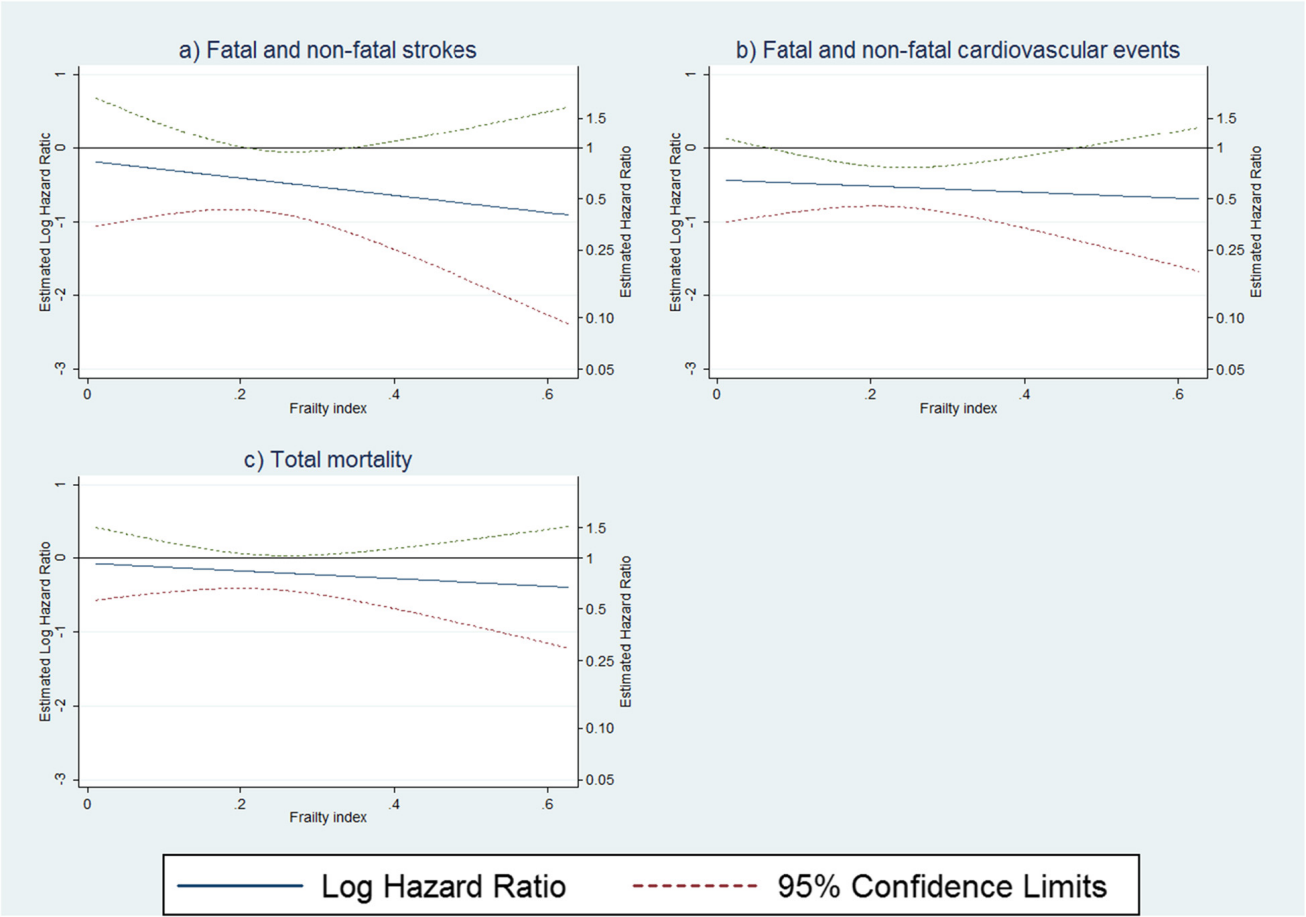
Estimates of the frailty specific log hazard ratio for treatment effect (active treatment versus placebo) and point-wise 95% confidence limits versus baseline frailty index, adjusted for age and sex and stratified by region of recruitment. (**a**) Fatal and non-fatal strokes. (**b**) Fatal and non-fatal cardiovascular events. (**c**) Total mortality.

There was no significant interaction between treatment effect and frailty for any of the three endpoints (*P* values for the interaction term in the interaction models for stroke, cardiovascular events, and total mortality were 0.52, 0.73, and 0.61, respectively). Estimates of the log HR for the treatment effect obtained from these interaction models are plotted versus FI, with the associated CIs (Figure 2) and HRs for selected values of FI (FI = 0.1, 0.2, 0.3, 0.4, 0.5, and 0.6) are reported in Table 3. For all three models the point estimate of the HR for the treatment effect decreases as FI increases, although to varying degrees and with varying certainty.

**Table 3:** **Estimated hazard ratios for treatment effect (active treatment versus placebo) and associated 95% confidence intervals, by frailty index**

	**Stroke**		**Cardiovascular events**		**Total mortality**	
**Frailty index**	**HR**	**95% CI**	**HR**	**95% CI**	**HR**	**95% CI**
0.1	0.75	0.40–1.38	0.62	0.42–0.92	0.89	0.63–1.25
0.2	0.66	0.43–1.01	0.60	0.45–0.78	0.84	0.66–1.07
0.3	0.59	0.36–0.96	0.57	0.42–0.79	0.80	0.61–1.04
0.4	0.52	0.25–1.09	0.55	0.34–0.89	0.76	0.50–1.14
0.5	0.47	0.16–1.33	0.53	0.26–1.06	0.72	0.40–1.29
0.6	0.41	0.10–1.65	0.50	0.20–1.27	0.68	0.32–1.48

All models adjusted by age, sex, and interaction between treatment and frailty index, and stratified by region of recruitment.

### Association between frailty and premature withdrawal

There were 347 patients for whom baseline FI was calculable but who withdrew from the study and were therefore censored in the analysis at the date of withdrawal. The distribution of baseline FI was not the same for those who withdrew and those who did not withdraw from the study (Mann–Whitney test, *P* <0.0001). The median FI was 0.19 (IQR, 0.13–0.27) in those who withdrew from the study and 0.16 (IQR, 0.11–0.24) in those who did not withdraw. The withdrawal rate in the least frail category (FI ≤0.10) was 9% compared to 17% in the frailest category (FI ≥0.35) and there was no difference in withdrawal rate between the treatment groups (14.38% placebo group vs. 14.90% active treatment group). Within the most frail category (FI ≥0.35), the withdrawal rate was 21% in the active treatment group (n = 99) compared to 14% (n = 94) in the placebo group, but this was not significant (*P* = 0.18, χ^2^ test).

## Discussion

Frailty as measured by the FI was a strong predictor of stroke, total mortality, and cardiovascular events in the HYVET trial, which is in agreement with multiple analyses from observational datasets [8-13]. We found no evidence of an interaction between baseline FI and treatment with antihypertensives on risk of stroke, death from all causes, or cardiovascular events in very elderly people. Furthermore, the burden of frailty amongst HYVET participants at baseline was similar to that seen in population studies [13-17].

Overall this suggests both that the HYVET population is more representative in terms of frailty than may have been supposed, and that benefits associated with blood pressure lowering treatment are accrued in both frailer and fitter older adults. These results would imply that frailty alone should not be used as a criteria for determining whether or not the treatment of an individual aged 80 and over with an antihypertensive to lower blood pressure to a goal of <150/80 mmHg is justified. Nevertheless, further work is required to fully characterize the benefit risk balance in this age group, with particular attention paid to the impact of any treatment adverse effects and/or a diagnosis of dementia, especially as there is epidemiological evidence to suggest that high blood pressure may not be harmful, and may even be beneficial, in the very elderly who are frail or have a functional disability [24-27]. In general, as the degree of frailty increases, so does the chance of functional impairment [28,29] or mobility impairment [30]. Furthermore, as the associations between risk factors and adverse outcomes may differ at extreme age and in frailty subgroups, and be dependent on risk factor change over time, a more nuanced approach to the interpretation of results may be required.

However, our results must be interpreted with caution. The number of items (at least 30) required to complete an FI meant that, of the 3,845 participants randomized, only the 2,656 (69%) who consented to complete an additional quality of life questionnaire had sufficient data. Nevertheless, it is reassuring that those for whom an FI was not calculable differed chiefly only in relation to cognition, scoring on average one point higher on the MMSE at entry, statistically but not clinically different. It is also possible that treatment with an anti-hypertensive may have affected participant withdrawals differentially, leading to bias in our estimate of the treatment effect. However, we found no difference in withdrawal rate between the treatment groups (14.38% placebo group vs. 14.90% active treatment group) and even amongst the frailest participants (FI ≥0.35) the withdrawal rate was only 17% overall. Although only 2,656 participants were available from the HYVET trial for these analyses these data still represent a significant number of older adults.

One must also be careful not to over interpret the results from the interaction models presented in Table 3 and Figure 2. Although the estimate of the log HR decreases as FI increases for all three endpoints, the interaction term in the respective models were not significant (overall the relationship between frailty and treatment effect was not strong). Furthermore, the wider CIs in the extremes are to be expected, given that very little of our data lies in the region FI <0.1 and FI >0.4 (Figure 2).

This analysis has some strengths. In particular, the use of data from a double-blind, placebo-controlled clinical trial also allows exploration of this by randomised group providing a more robust finding that would be possible from observational data alone. The similarity of the FI at baseline with large observational datasets supports the potential applicability of the results. As far as the authors know, this is the first time the FI has been used in analysing the results of a clinical trial, particularly a hypertensive very elderly group. Although only 69% of the randomized patients were included, this still constitutes a significant number within the literature base for this age group.

## Conclusions

Our analyses show that in the HYVET study participants there was no evidence of an interaction between treatment effect and frailty. Both the frailer and the fitter older adults with hypertension appeared to gain from treatment. Further work in is needed to confirm these findings in other similar datasets and to explore whether antihypertensive treatment modifies frailty as measured by the FI. Additionally, examining this more holistically, looking at impact of treatment over time, would be of additional benefit and is motivating further research by our group.

### Ethical approval

All appropriate ethical and regulatory permissions were obtained. This included approval in all participating countries by national drug or healthcare agencies, national ethics committees, and where required local ethical committees. The documentation is therefore large and complex. Full copies are archived by Imperial College London. HYVET was approved in the UK by the Multi-Centre Research Ethics Committee for Wales (MREC 98/9/16).

## Additional files

Additional file 1:
**Constituents of the Frailty Index.**
Additional file 2: Table S1. Variables included in the calculation of the Frailty Index (percent present in each treatment group).

## Abbreviations

CI: Confidence interval
FI: Frailty Index
HR: Hazard ratio
HYVET: HYpertension in the Very Elderly Trial
IQR: Interquartile range
MMSE: Mini-Mental State Exam
SD: Standard deviation
