# Supplementary material for: No evidence that frailty modifies the positive impact of antihypertensive treatment in very elderly people: an investigation of the impact of frailty upon treatment effect in the HYpertension in the Very Elderly Trial (HYVET) study, a double-blind, placebo-controlled study of antihypertensives in people with hypertension aged 80 and over
Source: BMC Med. 2015 Apr 9;13:78. doi: 10.1186/s12916-015-0328-1 (PMC4404571; doi:10.1186/s12916-015-0328-1)
Supplement: Additional file 1: — Constituents of the Frailty Index. [file 12916_2015_328_MOESM1_ESM.docx]

Additional file 1

Constituents of the frailty index (FI) (score 1 if the condition/deficit is present, 0 otherwise). The FI is calculated as the sum of the individual scores (components 1–60, listed below) divided by the number of non-missing scores. If the number of non-missing components is <30 then the FI is not calculable (i.e., set to missing).

| 1. | Diabetes (diagnosis of diabetes or use of a diabetic drug or a blood glucose of ≥7 mmol/l if fasting or a blood glucose of ≥11.1 mmol/l where non-fasting) |
| --- | --- |
| 2. | Previous cardiovascular disease (stroke, myocardial infarction, heart failure prior to entering the trial) |
| 3. | Overweight (BMI >24.9 and <30 if recruited outside China, >22.99 and <27.5 if recruited in China) |
| 4. | Underweight (BMI <18.5 regardless of region of recruitment) |
| 5. | Orthostatic hypotension (if systolic blood pressure falls >19 or diastolic blood pressure falls >9 mmHg) |
| 6. | Atrial fibrillation (reported as present after local investigator reviewed patient ECG) |
| 7. | Proteinuria (present/absent) |
| 8. | Haemoglobin (>18 or <10.5 gm/dl) |
| 9. | Potassium (>6 or <3.5 mmol/l) |
| 10. | Sodium (>150 or <135 mmol/l) |
| 11. | Haematocrit (<35%) |
| 12. | Urea (>7.5 or <3 mmol/l) |
| 13. | Uric Acid (>900 or <100 μmol/l) |
| 14. | Creatinine (>150 or <40 μmol/l) |
| 15. | Total cholesterol (>7 or <3.5 mmol/l) |
| 16. | HDL cholesterol (>1.5 or <0.4 mmol/l) |
| 17. | Glucose (>15 or <0.9 mmol/l) |
| 18. | Smoking (present/absent) |
| 19. | Systolic BP high (≥180 mmHg) |
| 20. | Diastolic BP high (≥90 mmHg) |
| 21. | SF36 question 3 “Does your health limit you in vigorous activities?” (answered ‘yes limited a lot’) |
| 22. | SF36 q4 “Does your health limit you in moderate activities?” (answered ‘yes limited a lot’) |
| 23. | SF36 q5 “Does your health limit you in lifting and carrying groceries?” (answered ‘yes limited a lot’) |
| 24. | SF36 q6 “Does your health limit you in climbing SEVERAL flights of stairs?” (answered ‘yes limited a lot’) |
| 25. | SF36 q7 “Does your health limit you in climbing ONE flight of stairs?” (answered ‘yes limited a lot’) |
| 26. | SF36 q8 “Does your health limit you in bending, kneeling, or stooping?” (answered ‘yes limited a lot’) |
| 27. | SF36 q9 “Does your health limit you in walking MORE THAN A MILE?” (answered ‘yes limited a lot’) |
| 28. | SF36 q10 “Does your health limit you in walking HALF A MILE?” (answered ‘yes limited a lot’) |
| 29. | SF36 q11 “Does your health limit you in walking 100 YARDS?” (answered ‘yes limited a lot’) |
| 30. | SF36 q12 “Does your health limit you in bathing and dressing yourself?” (answered ‘yes limited a lot’) |
| 31. | SF36 q13 “During the past 4 weeks have you cut down on the amount of time you spent on work or other activities?” (answered ‘yes’) |
| 32. | SF36 q15 “During the past 4 weeks were you limited in the kind of work or other activities?” (answered ‘yes’) |
| 33. | SF36 q16 “During the past 4 weeks have you had difficulty in performing the work or other activities (e.g., it took extra effort)?” (answered ‘yes’) |
| 34. | SF36 q20 “During the past 4 weeks, to what extent have your physical health or emotional problems interfered with your normal social activities with family, friends, neighbours, or groups?” (answered ‘quite a bit’ or ‘extremely’) |
| 35. | SF36 q21 “How much bodily pain have you had during the past 4 weeks?” (answered ‘severe’ or ‘very severe’) |
| 36. | SF36 q22 “During the past 4 weeks, how much did pain interfere with your normal work (including work both outside the home and housework)? (answered ‘quite a bit’ or ‘extremely’) |
| 37. | Orientation Memory Concentration (OMC) test question “Can the patient name the year?” (answered ‘no’) |
| 38. | OMC q2 “Can the patient name the month?” (answered ‘no’) |
| 39. | OMC q3 “Can the patient give the correct time (to within one hour)?” (answered ‘no’) |
| 40. | OMC q4 “Please count backwards from 20 subtracting 1 at a time” (if any errors) |
| 41. | OMC q5 “Please say the months of the year backwards” (if any errors) |
| 42. | OMC q6 “”Please repeat the name and address that I told you at the beginning of these questions” (if three or more errors when recalling an address given in a memory test) |
| 43. | Geriatric Depression Score (GDS) (>4) |
| 44. | GDS question “Have you dropped many of your activities and interests?” (answered ‘yes’) |
| 45. | GDS question “Do you prefer to stay at home rather than going out and doing new things?” (answered ‘yes’) |
| 46. | GDS question “Do you feel you have more problems with memory than most?” (answered ‘yes’) |
| 47. | GDS question “Do you feel full of energy?” (answered ‘no’) |
| 48. | Activities of daily living (ADL) (cannot wash without assistance) |
| 49. | ADL incontinent of urine |
| 50. | ADL cannot go out of the house and walk along the road without assistance |
| 51. | Any other impairment in ADL |
| 52. | Patient reported weak limbs (extremely bothered by the symptom in the last 4 weeks) |
| 53. | Patient reported blurred vision (extremely bothered by the symptom in the last 4 weeks) |
| 54. | Patient reported shortness of breath (extremely bothered by the symptom in the last 4 weeks) |
| 55. | Patient reported swollen ankles (extremely bothered by the symptom in the last 4 weeks) |
| 56. | Patient reported constipation (extremely bothered by the symptom in the last 4 weeks) |
| 57. | Patient reported bad taste in mouth (extremely bothered by the symptom in the last 4 weeks) |
| 58. | Patient reported racing heart (extremely bothered by the symptom in the last 4 weeks) |
| 59. | Patient reported cold hands or feet (extremely bothered by the symptom in the last 4 weeks) |
| 60. | Patient reported heart thumps or misses a beat (extremely bothered by the symptom in the last 4 weeks) |
