# Supplementary material for: No evidence that frailty modifies the positive impact of antihypertensive treatment in very elderly people: an investigation of the impact of frailty upon treatment effect in the HYpertension in the Very Elderly Trial (HYVET) study, a double-blind, placebo-controlled study of antihypertensives in people with hypertension aged 80 and over
Source: BMC Med. 2015 Apr 9;13:78. doi: 10.1186/s12916-015-0328-1 (PMC4404571; doi:10.1186/s12916-015-0328-1)
Supplement: Additional file 2: Table S1. — Variables included in the calculation of the Frailty Index (percent present in each treatment group). [file 12916_2015_328_MOESM2_ESM.docx]

Supplemental Table 1: Variables included in the calculation of the Frailty Index (percent present in each treatment group)

| No. | Variable | Placebo  (%) | Active Treatment  (%) |
| --- | --- | --- | --- |
| 1 | Diabetes | 10.4 | 9.8 |
| 2 | Previous cardiovascular disease | 12.0 | 11.5 |
| 3 | Overweight | 54.1 | 52.7 |
| 4 | Underweight | 2.5 | 3.8 |
| 5 | Orthostatic hypotension | 9.9 | 8.0 |
| 6 | Atrial fibrillation | 6.1 | 6.4 |
| 7 | Proteinuria (present/absent) | 12.9 | 12.6 |
| 8 | Haemoglobin (>18 or 9<10.5 gm/dl) | 5.9 | 5.5 |
| 9 | Potassium (>6 or <3.5 mmol/l) | 0.2 | 0.0 |
| 10 | Sodium (>150 or <135 mmol/l) | 5.8 | 6.0 |
| 11 | Haematocrit (<35%) | 12.8 | 13.8 |
| 12 | Urea (>7.5 or <3 mmol/l) | 22.4 | 23.3 |
| 13 | Uric Acid (>900 or <100 µmol/l) | 0.5 | 0.3 |
| 14 | Creatinine (>150 or <40 µmol/l) | 0.2 | 0.5 |
| 15 | Total cholesterol (>7 or <3.5 mmol/l) | 9.0 | 11.0 |
| 16 | HDL cholesterol (>1.5 or <0.4 mmol/l) | 23.4 | 23.6 |
| 17 | Glucose (>15 or <0.9 mmol/l) | 0.3 | 0.4 |
| 18 | Smoking (present/absent) | 6.7 | 6.5 |
| 19 | Systolic BP high (>=180mmHg) | 26.0 | 24.8 |
| 20 | Diastolic BP high (>=90mmHg) | 67.5 | 67.3 |
| 21 | SF36 question 3 “Does your health limit you in vigorous activities?” (answered ‘yes limited a lot’) | 52.8 | 53.6 |
| 22 | SF36 q4 “Does your health limit you in moderate activities?” (answered ‘yes limited a lot’) | 21.0 | 21.7 |
| 23 | SF36 q5 “Does your health limit you in lifting and carrying groceries?” (answered ‘yes limited a lot’) | 7.9 | 7.7 |
| 24 | SF36 q6 “Does your health limit you in climbing SEVERAL flights of stairs?” (answered ‘yes limited a lot’) | 30.2 | 30.2 |
| 25 | SF36 q7 “Does your health limit you in climbing ONE flight of stairs?” (answered ‘yes limited a lot’) | 8.7 | 8.0 |
| 26 | SF36 q8 “Does your health limit you in bending, kneeling or stooping?” (answered ‘yes limited a lot’) | 9.9 | 9.9 |
| 27 | SF36 q9 “Does your health limit you in walking MORE THAN A MILE?” (answered ‘yes limited a lot’) | 29.9 | 29.1 |
| 28 | SF36 q10 “Does your health limit you in walking HALF A MILE?” (answered ‘yes limited a lot’) | 12.2 | 13.7 |
| 29 | SF36 q11 “Does your health limit you in walking 100 YARDS?” (answered ‘yes limited a lot’) | 3.6 | 4.0 |
| 30 | SF36 q12 “Does your health limit you in bathing and dressing yourself?” (answered ‘yes limited a lot’) | 2.1 | 1.9 |
| 31 | SF36 q13 “During the past 4 weeks have you cut down on the amount of time you spent on work or other activities?” (answered ‘yes’) | 23.4 | 23.7 |
| 32 | SF36 q15 “During the past 4 weeks were you limited in the kind of work or other activities?” (answered ‘yes’) | 28.6 | 28.3 |
| 33 | SF36 q16 “During the past 4 weeks have you had difficulty in performing the work or other activities (e.g. it took extra effort)?” (answered ‘yes’) | 27.1 | 27.9 |
| 34 | SF36 q20 “During the past 4 weeks, to what extent has your physical health or emotional problems interfered with your normal social activities with family, friends, neighbours or groups?” (answered ‘quite a bit’ or ‘extremely’) | 6.9 | 5.7 |
| 35 | SF36 q21 “How much bodily pain have you had during the past 4 weeks?”(answered ‘severe’ or ‘very severe’) | 5.4 | 4.9 |
| 36 | SF36 q22 “During the past 4 weeks, how much did pain interfere with your normal work (including work both outside the home and housework)? (answered ‘quite a bit’ or ‘extremely’) | 8.1 | 7.6 |
| 37 | Orientation Memory Concentration test question “Can the patient name the year?” (answered ‘no’) | 6.8 | 7.3 |
| 38 | OMC q2 “Can the patient name the month?” (answered ‘no’) | 2.9 | 2.6 |
| 39 | OMC q3 “Can the patient give the correct time (to within one hour)?” (answered ‘no’) | 4.6 | 4.6 |
| 40 | OMC q4 “Please count backwards from 20 subtracting 1 at a time” (if any errors) | 12.8 | 12.0 |
| 41 | OMC q5 “Please say the months of the year backwards” (if any errors) | 13.4 | 12.4 |
| 42 | OMC q6 “”Please repeat the name and address that I told you at the beginning of these questions” (if three or more errors when recalling an address given in a memory test) | 7.4 | 7.2 |
| 43 | Geriatric Depression Score (>4) | 16.3 | 16.3 |
| 44 | GDS question “Have you dropped many of your activities and interests?” (answered ‘yes’) | 30.0 | 28.8 |
| 45 | GDS question “Do you prefer to stay at home rather than going out and doing new things?” (answered ‘yes’) | 32.7 | 32.7 |
| 46 | GDS question “Do you feel you have more problems with memory than most?” (answered ‘yes’) | 25.3 | 25.1 |
| 47 | GDS question “Do you feel full of energy?” (answered ‘no’) | 34.7 | 34.4 |
| 48 | Activities of daily living (ADL) (cannot wash without assistance) | 7.2 | 6.4 |
| 49 | ADL incontinent of urine | 6.2 | 6.7 |
| 50 | ADL cannot go out of the house and walk along the road without assistance | 7.1 | 7.0 |
| 51 | ADL any other impairment | 5.4 | 6.1 |
| 52 | Patient reported weak limbs | 6.7 | 7.1 |
| 53 | Patient reported blurred vision | 6.4 | 7.2 |
| 54 | Patient reported shortness of breath | 3.8 | 3.2 |
| 55 | Patient reported swollen ankles | 2.4 | 1.5 |
| 56 | Patient reported constipation | 4.8 | 4.9 |
| 57 | Patient reported bad taste in mouth | 1.8 | 2.0 |
| 58 | Patient reported racing heart | 2.7 | 1.7 |
| 59 | Patient reported cold hands or feet | 4.9 | 4.7 |
| 60 | Patient reported heart thumps or misses a beat | 2.2 | 1.4 |
